# Supplementary material for: Mechanisms governing the pioneering and redistribution capabilities of the non-classical pioneer PU.1
Source: Nat Commun. 2020 Jan 21;11:402. doi: 10.1038/s41467-019-13960-2 (PMC6972792; doi:10.1038/s41467-019-13960-2)
Supplement: Supplementary file 7 — Source data [file 41467_2019_13960_MOESM7_ESM.zip › Source_Data/FigureS2/FigureS2B_MotifScanOutput/KmeansCluster3/homerResults.html]

/misc/data/analysis/project\_PU1/CTV1/motifs/basic/PU1\_Kmeans14.3/final - Homer de novo Motif Results


# Homer *de novo* Motif Results (/misc/data/analysis/project\_PU1/CTV1/motifs/basic/PU1\_Kmeans14.3/final)

Known Motif Enrichment Results  
Gene Ontology Enrichment Results  
If Homer is having trouble matching a motif to a known motif, try copy/pasting the matrix file into
STAMP  
More information on motif finding results: HOMER
| Description of Results
| Tips
  
Total target sequences = 4769  
Total background sequences = 42408  
\* - possible false positive  

|  |  |  |  |  |  |  |  |  |
| --- | --- | --- | --- | --- | --- | --- | --- | --- |
| Rank | Motif | P-value | log P-pvalue | % of Targets | % of Background | STD(Bg STD) | Best Match/Details | Motif File |
| 1 | C G T A C G T A C G T A T C A G C T G A C T A G A C T G C G T A C G T A T A C G A C G T T A C G | 1e-2802 | -6.453e+03 | 87.15% | 10.10% | 26.0bp (79.4bp) | SpiB(ETS)/OCILY3-SPIB-ChIP-Seq(GSE56857)/Homer(0.962) More Information \| Similar Motifs Found | motif file (matrix) |
| 2 | G C T A A G T C A C G T G A C T T C G A A G T C C G A T | 1e-611 | -1.408e+03 | 59.51% | 21.60% | 57.8bp (70.9bp) | HOW(KH)/Drosophila\_melanogaster-RNCMPT00118-PBM/HughesRNA(0.764) More Information \| Similar Motifs Found | motif file (matrix) |
| 3 | C T G A A T G C G C T A C T A G C T A G T G C A C T G A A T G C T G A C T A C G | 1e-513 | -1.181e+03 | 31.70% | 6.30% | 49.5bp (72.6bp) | Ets1-distal(ETS)/CD4+-PolII-ChIP-Seq(Barski\_et\_al.)/Homer(0.818) More Information \| Similar Motifs Found | motif file (matrix) |
| 4 | T G C A C G T A T G C A T C A G T C A G A T C G C T G A C G T A A T G C | 1e-336 | -7.738e+02 | 40.05% | 14.93% | 51.3bp (73.4bp) | PB0058.1\_Sfpi1\_1/Jaspar(0.771) More Information \| Similar Motifs Found | motif file (matrix) |
| 5 | A C T G C T G A C T G A A C T G T C A G C G T A A C T G | 1e-255 | -5.876e+02 | 69.30% | 43.44% | 52.5bp (85.1bp) | RBM5(RRM)/Homo\_sapiens-RNCMPT00154-PBM/HughesRNA(0.959) More Information \| Similar Motifs Found | motif file (matrix) |
| 6 | T A G C C T G A A G T C A C G T A C G T T A G C G C A T T C A G G A T C G C T A | 1e-225 | -5.195e+02 | 19.67% | 5.23% | 50.3bp (73.7bp) | SpiB(ETS)/OCILY3-SPIB-ChIP-Seq(GSE56857)/Homer(0.782) More Information \| Similar Motifs Found | motif file (matrix) |
| 7 | C G A T C A T G A G T C G A C T G A C T T A G C G A T C G A C T A T G C G A C T A G C T A G T C | 1e-150 | -3.476e+02 | 22.86% | 9.15% | 49.0bp (90.2bp) | SPIC/MA0687.1/Jaspar(0.779) More Information \| Similar Motifs Found | motif file (matrix) |
| 8 | C G T A C T A G A C T G C T A G A C T G C T A G C G T A | 1e-123 | -2.832e+02 | 37.01% | 21.10% | 46.9bp (77.9bp) | RO3G\_00049(RRM)/Rhizopus\_oryzae-RNCMPT00205-PBM/HughesRNA(0.914) More Information \| Similar Motifs Found | motif file (matrix) |
| 9 | T C G A G A T C C G A T A G C T G A T C G A T C G T A C G A T C A G C T A C G T C A G T T A C G T A G C | 1e-122 | -2.821e+02 | 5.75% | 0.66% | 55.5bp (72.0bp) | SpiB(ETS)/OCILY3-SPIB-ChIP-Seq(GSE56857)/Homer(0.784) More Information \| Similar Motifs Found | motif file (matrix) |
| 10 | A G T C G A C T A G C T A G T C G A T C G A C T A G T C G A T C G A C T A G T C G A T C G A C T A G T C G A T C | 1e-109 | -2.531e+02 | 10.74% | 3.04% | 52.2bp (94.9bp) | ZNF263/MA0528.1/Jaspar(0.763) More Information \| Similar Motifs Found | motif file (matrix) |
| 11 | T C A G A T C G A T C G A C T G C T G A C G T A T A C G G A C T T G C A | 1e-94 | -2.171e+02 | 8.81% | 2.38% | 52.8bp (73.7bp) | RO3G\_00049(RRM)/Rhizopus\_oryzae-RNCMPT00205-PBM/HughesRNA(0.790) More Information \| Similar Motifs Found | motif file (matrix) |
| 12 | C T G A T A G C A T G C C A G T G A T C A G T C A G T C A G C T A G T C | 1e-86 | -1.994e+02 | 24.09% | 12.85% | 57.4bp (73.6bp) | PCBP2(KH)/Homo\_sapiens-RNCMPT00044-PBM/HughesRNA(0.742) More Information \| Similar Motifs Found | motif file (matrix) |
| 13 | G T C A T C G A A C T G A T C G G C T A C G T A A C T G A C G T C A T G G T C A C T G A C G T A | 1e-77 | -1.783e+02 | 3.12% | 0.27% | 45.5bp (65.4bp) | PU.1:IRF8(ETS:IRF)/pDC-Irf8-ChIP-Seq(GSE66899)/Homer(0.885) More Information \| Similar Motifs Found | motif file (matrix) |
| 14 | T G C A T C G A C T A G G T C A T G C A A T C G C G A T A C T G A T C G A G C T | 1e-69 | -1.612e+02 | 4.32% | 0.75% | 51.6bp (65.5bp) | ETS:RUNX(ETS,Runt)/Jurkat-RUNX1-ChIP-Seq(GSE17954)/Homer(0.765) More Information \| Similar Motifs Found | motif file (matrix) |
| 15 | C G T A T C A G C G A T C T A G T A C G G T C A C G T A C T A G C G A T T C A G T A G C C G A T C A T G C A T G | 1e-67 | -1.565e+02 | 2.70% | 0.23% | 51.8bp (49.0bp) | MOD(RRM)/Drosophila\_melanogaster-RNCMPT00140-PBM/HughesRNA(0.730) More Information \| Similar Motifs Found | motif file (matrix) |
| 16 | G T A C C G T A A T C G A G C T A C G T A G T C A G T C C G A T T A G C C G T A C T A G C T G A T C G A C T G A | 1e-62 | -1.436e+02 | 6.12% | 1.72% | 48.9bp (66.4bp) | PU.1(ETS)/ThioMac-PU.1-ChIP-Seq(GSE21512)/Homer(0.764) More Information \| Similar Motifs Found | motif file (matrix) |
| 17 | T C G A A G T C G A C T A C G T A G T C G A T C G C A T C A T G A C T G A G T C G C A T A T C G A G C T | 1e-59 | -1.374e+02 | 9.14% | 3.53% | 48.8bp (73.9bp) | Eip74EF/dmmpmm(Bigfoot)/fly(0.769) More Information \| Similar Motifs Found | motif file (matrix) |
| 18 | G T C A G T C A A G T C G A C T C A G T C T G A T A G C G C T A T C G A C A G T A G T C T G C A | 1e-53 | -1.241e+02 | 2.33% | 0.23% | 70.7bp (69.9bp) | Dux/MA0611.1/Jaspar(0.669) More Information \| Similar Motifs Found | motif file (matrix) |
| 19 | G A C T C A G T A T G C T A G C G A C T G A T C T G C A G T C A G C T A T C G A | 1e-47 | -1.094e+02 | 5.51% | 1.76% | 55.3bp (62.9bp) | CEBP(bZIP)/ThioMac-CEBPb-ChIP-Seq(GSE21512)/Homer(0.708) More Information \| Similar Motifs Found | motif file (matrix) |
| 20 | C A T G A C G T A C G T C A G T C T G A C T G A C T G A T C G A C T A G C G A T C A T G A C G T C T A G | 1e-47 | -1.090e+02 | 1.95% | 0.18% | 30.8bp (69.0bp) | Unknown6/Drosophila-Promoters/Homer(0.675) More Information \| Similar Motifs Found | motif file (matrix) |
| 21 | A T G C C G T A C T A G G C A T A C G T A G T C A C G T A G T C T C G A A G C T A C T G C G T A C A T G C G T A | 1e-47 | -1.086e+02 | 3.17% | 0.61% | 43.6bp (73.6bp) | ZBTB33(Zf)/GM12878-ZBTB33-ChIP-Seq(GSE32465)/Homer(0.724) More Information \| Similar Motifs Found | motif file (matrix) |
| 22 | G T A C A G C T G A T C C G A T G C A T T C A G C T G A C T A G C T G A T G C A A T G C A C G T G T A C G T C A | 1e-41 | -9.516e+01 | 3.02% | 0.64% | 55.5bp (69.6bp) | EXC-7(RRM)/Caenorhabditis\_elegans-RNCMPT00014-PBM/HughesRNA(0.652) More Information \| Similar Motifs Found | motif file (matrix) |
| 23 | A C T G C G A T A T G C A C T G C G T A C G T A C T G A A C G T | 1e-40 | -9.395e+01 | 8.47% | 3.84% | 47.9bp (69.3bp) | DDF1(AP2EREBP)/col-DDF1-DAP-Seq(GSE60143)/Homer(0.700) More Information \| Similar Motifs Found | motif file (matrix) |
| 24 | T A C G T C G A C T G A A G T C G A C T T G A C G C T A A G C T G A C T T A G C G C T A A T G C | 1e-37 | -8.529e+01 | 1.97% | 0.28% | 61.0bp (67.4bp) | TARDBP(RRM)/Homo\_sapiens-RNCMPT00076-PBM/HughesRNA(0.720) More Information \| Similar Motifs Found | motif file (matrix) |
| 25 | C G T A A T G C C G A T A G C T G T A C G A T C T A G C A G T C C T G A C G T A C G T A | 1e-36 | -8.406e+01 | 3.25% | 0.84% | 51.1bp (68.1bp) | PB0058.1\_Sfpi1\_1/Jaspar(0.655) More Information \| Similar Motifs Found | motif file (matrix) |
| 26 | T C G A A C T G A G T C C G T A A C T G C G T A C T G A A T C G A G T C | 1e-35 | -8.121e+01 | 5.47% | 2.12% | 50.8bp (71.7bp) | POL008.1\_DCE\_S\_I/Jaspar(0.683) More Information \| Similar Motifs Found | motif file (matrix) |
| 27 | A C G T A G T C A G T C A G C T C T A G C G T A A C T G T C A G A G T C A G T C C G A T A G T C G A T C | 1e-35 | -8.106e+01 | 1.74% | 0.22% | 78.7bp (70.4bp) | AP-2alpha(AP2)/Hela-AP2alpha-ChIP-Seq(GSE31477)/Homer(0.633) More Information \| Similar Motifs Found | motif file (matrix) |
| 28 | C T A G C T G A G A C T G A C T A T C G C T A G C T G A A G C T A G T C C T G A A G C T | 1e-34 | -7.983e+01 | 1.61% | 0.19% | 73.2bp (63.2bp) | AARE(HLH)/mES-cMyc-ChIP-Seq/Homer(0.806) More Information \| Similar Motifs Found | motif file (matrix) |
| 29 | A C T G C G T A C T A G G C A T C G T A G A T C A C G T T A G C G C T A G A C T | 1e-34 | -7.953e+01 | 2.37% | 0.47% | 50.3bp (67.8bp) | PB0152.1\_Nkx3-1\_2/Jaspar(0.713) More Information \| Similar Motifs Found | motif file (matrix) |
| 30 | A G T C C G A T A G T C T C G A A G T C A G C T C T G A A G C T G T A C C G T A A G T C T C A G C T G A T A C G | 1e-34 | -7.854e+01 | 1.78% | 0.25% | 48.9bp (55.6bp) | FZF1/MA0298.1/Jaspar(0.653) More Information \| Similar Motifs Found | motif file (matrix) |
| 31 | C T G A A G C T G A T C G T A C C T G A T C G A A G C T G T A C C G T A A G T C A G T C G A C T A G T C A G T C | 1e-34 | -7.854e+01 | 1.20% | 0.08% | 78.7bp (52.2bp) | HAP4(MacIsaac)/Yeast(0.638) More Information \| Similar Motifs Found | motif file (matrix) |
| 32 | T G C A G A T C C A T G C T G A T C A G T G C A C G T A A T G C C G T A T A C G A G T C | 1e-30 | -7.013e+01 | 3.06% | 0.88% | 57.1bp (72.9bp) | Ot\_0263(RRM)/Ostreococcus\_tauri-RNCMPT00263-PBM/HughesRNA(0.760) More Information \| Similar Motifs Found | motif file (matrix) |
| 33 | A C T G A G T C C G T A A C T G C T A G G T C A A C T G C G T A C T A G C G T A A T C G C G T A A C T G | 1e-28 | -6.528e+01 | 1.09% | 0.09% | 39.8bp (72.2bp) | SeqBias: GA-repeat(0.768) More Information \| Similar Motifs Found | motif file (matrix) |
| 34 | A G T C C G A T A G C T G A T C A G C T A G C T G T A C G C T A A G T C C T G A C G A T C T A G C T A G A G T C | 1e-27 | -6.393e+01 | 1.80% | 0.34% | 42.5bp (76.1bp) | INO4/INO4\_YPD/4-INO4,37-INO2(Harbison)/Yeast(0.803) More Information \| Similar Motifs Found | motif file (matrix) |
| 35 | A G T C A G C T A G C T G T A C A G C T C T A G T G A C G A T C T C G A G A C T | 1e-26 | -6.183e+01 | 5.33% | 2.36% | 57.2bp (68.7bp) | schlank/MA0193.1/Jaspar(0.699) More Information \| Similar Motifs Found | motif file (matrix) |
| 36 | C G T A C G T A A C T G A C G T A C T G A C G T A C T G | 1e-26 | -6.137e+01 | 6.96% | 3.49% | 48.0bp (69.5bp) | RBM24(RRM)/Homo\_sapiens-RNCMPT00184-PBM/HughesRNA(0.848) More Information \| Similar Motifs Found | motif file (matrix) |
| 37 | A C G T A C G T A G T C A G T C A G C T A C T G G T A C A C T G C T A G A G T C | 1e-26 | -6.134e+01 | 2.05% | 0.47% | 74.2bp (65.5bp) | PHD1/PHD1\_BUT90/8-SUT1(Harbison)/Yeast(0.736) More Information \| Similar Motifs Found | motif file (matrix) |
| 38 | G A C T C G A T G C T A G C T A G C T A G A T C G T A C G T C A A G C T A T G C C T G A | 1e-26 | -6.077e+01 | 2.83% | 0.86% | 50.0bp (59.8bp) | HuR(RRM)/Homo\_sapiens-RNCMPT00136-PBM/HughesRNA(0.666) More Information \| Similar Motifs Found | motif file (matrix) |
| 39 | A G C T G A T C C G T A A C G T A C T G C G T A C T A G C T G A C G T A G A T C G A C T A G T C C G T A G T A C | 1e-25 | -5.981e+01 | 0.86% | 0.05% | 58.5bp (7.6bp) | PB0194.1\_Zbtb12\_2/Jaspar(0.651) More Information \| Similar Motifs Found | motif file (matrix) |
| 40 | A C T G A T G C A C T G C G T A C G T A T A C G A G C T C G T A | 1e-25 | -5.832e+01 | 5.33% | 2.43% | 55.9bp (69.9bp) | SWI4/MA0401.1/Jaspar(0.705) More Information \| Similar Motifs Found | motif file (matrix) |
| 41 | A G T C C G T A C T A G A C T G A C T G C G T A C G T A A C T G A C G T C T A G | 1e-25 | -5.767e+01 | 1.89% | 0.42% | 40.9bp (68.3bp) | SD0002.1\_at\_AC\_acceptor/Jaspar(0.747) More Information \| Similar Motifs Found | motif file (matrix) |
| 42 | A C T G C G T A A C G T A C G T C A G T A G T C A G T C G T A C A G T C A G T C C G A T A C G T | 1e-25 | -5.760e+01 | 0.67% | 0.02% | 50.4bp (32.9bp) | NDD1(MacIsaac)/Yeast(0.746) More Information \| Similar Motifs Found | motif file (matrix) |
| 43 | T C A G A G T C C T G A C T A G A T G C G C T A T C G A T C A G T A G C T G C A | 1e-24 | -5.662e+01 | 20.47% | 14.58% | 55.3bp (70.5bp) | MBNL1(Znf)/Homo\_sapiens-RNCMPT00038-PBM/HughesRNA(0.748) More Information \| Similar Motifs Found | motif file (matrix) |
| 44 | C T G A C G A T G T C A C T A G A G C T A C T G C G T A A T C G A G C T C T A G C G T A | 1e-23 | -5.456e+01 | 1.34% | 0.21% | 50.2bp (71.6bp) | z/dmmpmm(Down)/fly(0.689) More Information \| Similar Motifs Found | motif file (matrix) |
| 45 | G T C A A C G T G T A C C T G A G A C T C T A G T C A G T C A G T C A G C A T G | 1e-22 | -5.114e+01 | 2.22% | 0.64% | 69.5bp (65.3bp) | Tv\_0259(RRM)/Trichomonas\_vaginalis-RNCMPT00259-PBM/HughesRNA(0.710) More Information \| Similar Motifs Found | motif file (matrix) |
| 46 | G T C A G T A C G T C A A C G T T A G C G A T C G A C T G T A C G C A T C A G T A G C T A G C T | 1e-21 | -5.005e+01 | 0.88% | 0.08% | 46.3bp (63.2bp) | SPIC/MA0687.1/Jaspar(0.736) More Information \| Similar Motifs Found | motif file (matrix) |
| 47 | G T C A A C T G C G T A G C T A A G T C C T A G C A T G A G T C G T C A | 1e-21 | -4.926e+01 | 2.35% | 0.73% | 55.2bp (76.3bp) | Lm\_0254(RRM)/Leishmania\_major-RNCMPT00254-PBM/HughesRNA(0.723) More Information \| Similar Motifs Found | motif file (matrix) |
| 48 | G T C A A C G T G T A C A C G T A T C G A C T G A C G T A C G T C T A G A C G T | 1e-20 | -4.635e+01 | 1.20% | 0.20% | 35.5bp (67.5bp) | grh/dmmpmm(Bigfoot)/fly(0.745) More Information \| Similar Motifs Found | motif file (matrix) |
| 49 | A G T C C T A G A C G T C T A G A C T G A G T C C T G A A C T G C G T A | 1e-19 | -4.568e+01 | 3.54% | 1.49% | 61.1bp (67.4bp) | bZIP68/MA0968.1/Jaspar(0.689) More Information \| Similar Motifs Found | motif file (matrix) |
| 50 | G C T A C G T A C G T A A G T C G C A T A C G T A T G C G T A C A G C T C G A T A T C G A G T C | 1e-19 | -4.530e+01 | 3.29% | 1.34% | 54.8bp (69.4bp) | ELF3(ETS)/PDAC-ELF3-ChIP-Seq(GSE64557)/Homer(0.681) More Information \| Similar Motifs Found | motif file (matrix) |
| 51 | C T A G A G T C A G T C C T G A C G A T C T A G A G C T C T A G T C G A | 1e-19 | -4.460e+01 | 9.31% | 5.76% | 56.7bp (66.7bp) | USF2/MA0526.1/Jaspar(0.725) More Information \| Similar Motifs Found | motif file (matrix) |
| 52 | G T A C A C G T A T G C A G T C G T A C C G T A G T A C G T A C G T C A A T C G A C T G A G T C | 1e-17 | -4.105e+01 | 1.05% | 0.18% | 77.0bp (65.5bp) | ZNF143\|STAF(Zf)/CUTLL-ZNF143-ChIP-Seq(GSE29600)/Homer(0.730) More Information \| Similar Motifs Found | motif file (matrix) |
| 53 | A C G T A C G T G T C A C T G A C G T A C G T A A C T G A G T C A C T G | 1e-16 | -3.811e+01 | 1.66% | 0.48% | 43.7bp (61.9bp) | KHDRBS1(KH)/Mus\_musculus-RNCMPT00062-PBM/HughesRNA(0.767) More Information \| Similar Motifs Found | motif file (matrix) |
| 54 | A G T C C G T A A T C G A G T C C G T A C T A G A T C G A C T G C T A G C T A G | 1e-15 | -3.510e+01 | 1.57% | 0.47% | 54.0bp (63.0bp) | Unknown-ESC-element(?)/mES-Nanog-ChIP-Seq(GSE11724)/Homer(0.885) More Information \| Similar Motifs Found | motif file (matrix) |
| 55 | G T C A A C G T A T C G C T G A C T A G C T A G T G C A C T G A C T A G C G T A A G T C A C G T | 1e-14 | -3.238e+01 | 1.40% | 0.41% | 56.9bp (70.7bp) | GCR1/Literature(Harbison)/Yeast(0.666) More Information \| Similar Motifs Found | motif file (matrix) |
| 56 | A G C T A G C T C G A T G T C A C G A T A T G C C G T A T C A G C G T A A C G T A G T C A G C T | 1e-14 | -3.225e+01 | 0.36% | 0.01% | 61.0bp (44.9bp) | GATA14(C2C2gata)/col-GATA14-DAP-Seq(GSE60143)/Homer(0.775) More Information \| Similar Motifs Found | motif file (matrix) |
| 57 | A T G C C G T A C G T A C G T A A G T C T G C A A C T G A G T C T G C A | 1e-13 | -3.150e+01 | 5.96% | 3.58% | 58.8bp (67.7bp) | RUNX2/MA0511.2/Jaspar(0.759) More Information \| Similar Motifs Found | motif file (matrix) |
| 58 | G T A C A C G T C T A G T G A C G A C T G C A T A G T C C G A T C A T G G A T C A G T C C G T A | 1e-13 | -3.130e+01 | 0.75% | 0.12% | 58.3bp (50.2bp) | Adf1/dmmpmm(Bergman)/fly(0.703) More Information \| Similar Motifs Found | motif file (matrix) |
| 59 | A C T G C G T A C G T A C G T A A G T C A C G T A C G T C G T A | 1e-13 | -3.043e+01 | 2.26% | 0.94% | 69.9bp (72.2bp) | EXC-7(RRM)/Caenorhabditis\_elegans-RNCMPT00014-PBM/HughesRNA(0.806) More Information \| Similar Motifs Found | motif file (matrix) |
| 60 | G A T C A G C T T C G A A C G T A C T G A C G T A C G T A C G T G T C A T G C A T A G C | 1e-12 | -2.906e+01 | 0.73% | 0.13% | 48.1bp (57.5bp) | AT3G25990(Trihelix)/colamp-AT3G25990-DAP-Seq(GSE60143)/Homer(0.759) More Information \| Similar Motifs Found | motif file (matrix) |
| 61 | A G C T C G T A C G T A A T G C G T A C C T G A A T G C | 1e-12 | -2.886e+01 | 12.16% | 8.85% | 57.4bp (67.9bp) | lbe/MA0231.1/Jaspar(0.854) More Information \| Similar Motifs Found | motif file (matrix) |
| 62 | A G T C A C G T A G T C A C T G G T A C A T C G A C G T A C T G A G T C | 1e-12 | -2.871e+01 | 3.98% | 2.17% | 48.5bp (77.0bp) | tin/MA0247.2/Jaspar(0.675) More Information \| Similar Motifs Found | motif file (matrix) |
| 63 | A C T G G T A C G A C T C T G A C T A G C G T A A C T G A G T C C G T A | 1e-12 | -2.769e+01 | 2.98% | 1.47% | 57.4bp (75.1bp) | ZNF415(Zf)/HEK293-ZNF415.GFP-ChIP-Seq(GSE58341)/Homer(0.764) More Information \| Similar Motifs Found | motif file (matrix) |
